# Gene Expression and Characterization of Iturin A Lipopeptide Biosurfactant from *Bacillus aryabhattai* for Enhanced Oil Recovery

**DOI:** 10.3390/gels8070403

**Published:** 2022-06-25

**Authors:** Deepak A. Yaraguppi, Zabin K. Bagewadi, Nilkamal Mahanta, Surya P. Singh, T. M. Yunus Khan, Sanjay H. Deshpande, Chaitra Soratur, Simita Das, Dimple Saikia

**Affiliations:** 1 Department of Biotechnology, KLE Technological University, Hubballi 580031, India; deepak.yaraguppi@gmail.com (D.A.Y.); sanjay.deshpande2389@gmail.com (S.H.D.); chaitrasoratur@gmail.com (C.S.); 2 Department of Chemistry, Indian Institute of Technology, Dharwad 580011, India; 203041001@iitdh.ac.in; 3 Department of Biosciences and Bioengineering, Indian Institute of Technology, Dharwad 580011, India; ssingh@iitdh.ac.in (S.P.S.); 202041002@iitdh.ac.in (D.S.); 4 Department of Mechanical Engineering, College of Engineering, King Khalid University, Abha 61421, Saudi Arabia; yunus.tatagar@gmail.com

**Keywords:** iturin A, lipopeptide, biosurfactant, expression, MALDI-TOF, Raman spectroscopy, enhanced oil recovery

## Abstract

Biosurfactants are eco-friendly surface-active molecules recommended for enhanced oil recovery techniques. In the present study, a potential lipopeptide (biosurfactant) encoding the iturin A gene was synthesized from *Bacillus aryabhattai*. To improvise the yield of the lipopeptide for specific applications, current research tends toward engineering and expressing recombinant peptides. An iturin A gene sequence was codon-optimized, amplified with gene-specific primers, and ligated into the pET-32A expression vector to achieve high-level protein expression. The plasmid construct was transformed into an *E. coli* BL21 DE3 host to evaluate the expression. The highly expressed recombinant iturin A lipopeptide was purified on a nickel nitrilotriacetic acid (Ni-NTA) agarose column. Sodium dodecyl sulfate polyacrylamide gel electrophoresis (SDS-PAGE) revealed that the purity and molecular mass of iturin A was 41 kDa. The yield of recombinant iturin A was found to be 60 g/L with a 6.7-fold increase in comparison with our previously published study on the wild strain. The approach of cloning a functional fragment of partial iturin A resulted in the increased production of the lipopeptide. When motor oil was used, recombinant protein iturin A revealed a biosurfactant property with a 74 ± 1.9% emulsification index (E24). Purified recombinant protein iturin A was characterized by mass spectrometry. MALDI-TOF spectra of trypsin digestion (protein/trypsin of 50:1 and 25:1) showed desired digested mass peaks for the protein, further confirming the identity of iturin A. The iturin A structure was elucidated based on distinctive spectral bands in Raman spectra, which revealed the presence of a peptide backbone and lipid. Recombinant iturin A was employed for enhanced oil recovery through a sand-packed column that yielded 61.18 ± 0.85% additional oil. Hence, the novel approach of the high-level expression of iturin A (lipopeptide) as a promising biosurfactant employed for oil recovery from *Bacillus aryabhattai* is not much reported. Thus, recombinant iturin A demonstrated its promising ability for efficient oil recovery, finding specific applications in petroleum industries.

## 1. Introduction

Biosurfactants are produced as extracellular molecules on microbial cell surfaces. These are surface-active molecules that reduce the surface tension between hydrophobic and hydrophilic regions and are produced by several microbes, such as bacteria, fungi, and yeast [1]. Biosurfactants are biochemically made up of hydrophilic segments, such as carbohydrates, polar peptides, or acids, and hydrophobic segments, usually lipids [2]. A lipopeptide is a specific class of biosurfactant that has a lipid moiety attached to a peptide and is produced as part of microbial metabolism. Lipopeptides have a wide range of applications, especially those produced by *Bacillus* species, which have been shown to exhibit antibacterial and antifungal activities [3]. Lipopeptides produced by bacterial species can be classified into three types, namely, surfactin, iturin, and fengycin. Out of these three lipopeptides, iturin is comparatively smaller and is amphiphilic in nature. It has a peptide linked to the fatty acid chain. As lipopeptides are molecules of biological origin, they are biodegradable, nontoxic, and nonpolluting biomolecules; hence. they are environmentally acceptable [4].

Iturin A is a type of cyclic lipopeptide produced by *Bacillus* species as a secondary molecule and acts as an antibiotic [5]. Iturin A has been known to show inhibitory activity by limiting the growth of fungi [6]. The lipopeptide nature of the biosurfactant has shown thermal, pH, and salinity stability [7]. Many researchers are interested in replacing synthetic surfactants with biosurfactants; however, a genuine problem still exists in the economics of production, lower yield, and costly downstream and recovery process of a biosurfactant [8]. However, with the application of statistical design methods, such as response surface methodology (RSM), the yields of biosurfactant production has improved by 2.51-fold using crude oil [7]. To reduce the cost involved in the production of biosurfactants, it is essential to construct hyperproducing or mutant strains with better yields [8]. Improved yield combined with a cost-effective carbon source can result in lesser production cost [9].

The development of recombinant biosurfactants is gaining scientific attention in recent times due to industrial applications. A strategy for improving the gene expression that encodes a biosurfactant is receiving focused interest to improve the yields and modify the chemical attributes [10]. The mycosubtilin and iturin A operons have been determined as operons of the iturin lipopeptide family. The *lpa-14* gene is a homologue of the *sfp* gene, which is responsible for the biosynthesis of surfactin and iturin A in *B. subtilis* RB14 [11]. The *Escherichia coli* lac operator and the promoter core region of the penicillinase gene of *B. licheniformis* present in the hybrid promoter P spac was reported to increase lipopeptide biosynthesis fivefold [12]. Biosurfactants are amphipathic molecules that enable them to perform a vital function. Recently, it has been proved that biosurfactants can solubilize and mobilize organic compounds adsorbed on soil constituents. Biosurfactants have shown good results for the decontamination of soil, as it has unique properties [13]. Biosurfactants can also be employed in biomedical applications owing to their antimicrobial and antiviral activities [14]. The lipopeptide surfactin is one of the most well-known and studied surfactants [15]. A lipopeptide can be used for antimycoplasmal, antibacterial, and antiviral activities. It can also be used for antiadhesive and anti-inflammatory applications. It has also shown good results for environmental applications, such as oil recovery, in environmental bioremediation, and it also accelerates the biodegradation of hydrocarbons [16]. With the help of genome shuffling, 179.22 mg/L of iturin A was obtained from *Bacillus amyloliquefaciens*, which had a 2.03-fold increase compared with a wild strain [17]. Iturin A production was 330 µg/mL from the recombinant *Bacillus subtilis* strain as compared with 110 µg/mL from a wild strain, which indicated a threefold increase [6,17]. A biosurfactant produced from *Bacillus* species was characterized previously by mass spectrometry and Raman spectroscopy [17,18].

However, not many reports are available on the high-level expression of iturin A (lipopeptide) as a promising biosurfactant from *Bacillus aryabhattai*. The current study reports the design and synthesis of the *lpa-14* gene responsible for iturin A production and cloning in the pET-32A vector, followed by the overexpression in *E. coli* BL21 DE3. The structural characterization of a purified recombinant iturin A protein was carried out by Raman spectroscopy, and molecular mass was analyzed by mass spectroscopy (MALDI-TOF MS). The biosurfactant activity of expressed iturin A was demonstrated in a sand-packed column for enhanced oil recovery.

## 2. Materials and Methods

### 2.1. Chemicals, Vectors, and Bacterial Strains

All the chemicals and nickel nitrilotriacetic acid (Ni-NTA) resin column used in the present research were purchased from Sigma-Aldrich Pvt Ltd. (Burlington, MA, USA) and Merck and Co. Inc. (Rahway, NJ, USA). The iturin A gene was synthesized from *Bacillus aryabhattai*. The pET-32A vector, pUC19 vector, *E. coli* BL21 DE3, and *E. coli* DH5α were used for cloning, sequencing, and expression activities.

### 2.2. Genome Sequence Identification

Iturin A production by *B. aryabhattai* was identified based on the results obtained from antiSMASH, which predicts the genome clusters producing secondary metabolites [18]. Iturin A synthesis is assisted by the enzyme phosphopantetheinyl transferase coded by the *lpa-14* gene. The FASTA sequence of the *lpa-14* gene was retrieved from the National Center Biotechnology Information (NCBI).

#### Gene Synthesis, Amplification, and Cloning

From the synthesized codon-optimized DNA template, the iturin A gene was amplified using the gene-specific primers and ligated into the pET-32A expression vector. The ligated product was transformed into an *E. coli* DH5α host. Using BamHI and EcoRI, restriction digestion was carried out for the confirmation of the gene insert. The FASTA sequence of the iturin A gene was retrieved from the NCBI (Accession no. QHD44466.1) database, and then it was reverse-translated using the Transeq tool, European Molecular Biology Laboratory (EMBL) [19]. The translated protein sequences were further analyzed for the presence of GC content and subjected to codon optimization for *E. coli* expression by interchanging certain nucleotide sequences without altering the protein sequence for the enhanced expression of the protein. The gene was amplified using synthesized T7 gene-specific primers (forward primer 5′-ATGAAAATTTACGGAGTATA-3′ and reverse primer 5′-TTATAACAGCTCTTCATACGTT-3′) from the synthesized DNA template. The amplified gene was cloned into the pUC19 vector.

Upon restriction digestion, the cloned insert was confirmed through sequencing. Further, the restricted insert was subcloned into the pET-32A bacterial expression vector; for this activity, BamHI and EcoRI with GGATCC and GAATTC restriction sites were employed. The pET-32A vector used for cloning was designed for the high-level expression of recombinant peptide sequences and was fused with a 109 amino acid thioredoxin protein tag, with cleavable His-tag and S-tag. The plasmid construct with a concentration of 106 ng/µL was transformed into *E. coli* BL21 DE3 chemical competent cells using heat shock to evaluate the expression. The transformants were subjected to lysate/colony PCR using gene-specific primers to confirm the presence of the insert (iturin A gene). The positive transformants were grown in Luria–Bertani (LB) media and evaluated for protein expression.

### 2.3. Expression of the Iturin A Lipopeptide

The pET-32A-vector-transformed fresh colony was selected and cultured on an LB agar plate. The starter culture was prepared by inoculating the culture into an LB medium containing ampicillin at 100 µg/mL. The medium was incubated at 37 ± 2 °C under shaking conditions (160 rpm) for 16 h. An amount of 10 mL of starter culture was added to the flask containing a 1 L LB medium with ampicillin and incubated at 37 ± 2 °C. The flask was kept under shaking conditions (200 rpm) to achieve optical density (OD) between 0.55 and 0.7 at 600 nm. Further, the flask was cooled to 16 °C, and isopropyl β-D-1-thiogalactopyranoside (IPTG) at 1 mM was added. The flask was kept at 15 °C at 180 rpm for 16–18 h. The induced cell culture was centrifuged at 4000 rpm at 4 °C for 20 min, and the pellet was collected and stored at −80 °C until further use. For protein purification, the cell pellet was resuspended in ice-cold lysis buffer (20 mM Na_2_HPO_4_, 500 mM NaCl, 10 mM imidazole, 2.5% *v*/*v* glycerol, and 0.1% *v*/*v* Triton), and to this, 80 mg of lysozyme, 20 µL of benzamidine, and 20 µL of phenylmethylsulfonyl fluoride (PMSF) were added. The solution was subjected to sonication for 1 min at 60 amplitude, and this cycle was repeated six times. The solution was centrifuged at 4900 rpm for 60 min at 4 °C. The obtained supernatant was collected in 2 mL tubes and centrifuged again at 4 °C at 11,000 rpm for 60 min to remove any precipitate or suspended material. The clear supernatant was further subjected to purification.

### 2.4. Purification of the Recombinant Iturin A Lipopeptide

For purification, the obtained supernatant containing protein was loaded onto an agarose column containing nickel nitrilotriacetic acid (Ni-NTA) resin pretreated with lysis buffer for purification of the His-tagged iturin A recombinant protein. The column was washed with 30 mL of wash buffer (20 mM Na_2_HPO_4_, 500 mM NaCl, 30 mM imidazole, 2.5% *v*/*v* glycerol, pH 7.40) to remove any nontagged proteins. An elution buffer (20 mM Na_2_HPO_4_, 300 mM NaCl, 300 mM imidazole, 2.5% *v*/*v* glycerol, pH 7.40) was used to elute the His-tagged recombinant protein from the column. The purity of the protein was assessed by sodium dodecyl sulfate polyacrylamide gel (12.5%) electrophoresis (SDS-PAGE) [8]. The expressed iturin A concentration was determined by the Bradford assay (using BSA as the standard protein). The absorbance measurement was recorded at A_280_ nm (molar extinction coefficient was obtained from the Expasy ProtParam tool using the primary sequence of iturin A; http://web.expasy.org/protparam/, accessed on 15 October 2021).

### 2.5. Evaluation of Biosurfactant Activity

Iturin A was examined for activities related to biosurfactants by various assays. The oil spread assay was carried out according to the method of Yaraguppi et al. (2020) [7]. An amount of 40 mL of distilled water was taken in a Petri plate, and to this, 25 µL of motor oil was added. An amount of 10 µL of purified recombinant iturin A was added to the oil surface, and oil displacement was measured. The dispersion index is calculated by measuring the diameter of oil after dispersion, and the oil dispersion area was calculated by using the equation $=\pi r^{2}$, where *r* is the radius. The biosurfactant activity of iturin A was also assessed by measuring the emulsification index (E24) [7]. Briefly, 3 mL of a recombinant protein (cell-free supernatant) was added to 3 mL motor oil, mixed rigorously (10 min) and allowed to stand for 24 h. The height of the emulsion layer (mm) divided by the total height of the solution (mm) multiplied by 100 indicated the percentage emulsification index [20].

### 2.6. Analytical Characterization

#### 2.6.1. Mass Spectrometry Analysis

To confirm the identity of the recombinant protein, mass spectrometric (MS) analysis was performed on a tryptic digest of iturin A. The digestion of protein was carried out by using Trypsin-ultra™ Mass Spectrometry Grade from New England Biolabs (NEB, Hertfordshire, UK). Two trypsin digestion reactions for the protein were set up with a protein/trypsin ratio of 50:1 and 25:1 each containing 1 µL of trypsin (1 µg/µL), 2.5 µL of 2X trypsin reaction buffer, 2.5 µL of ultrapurified water, and 50 and 25 µL of protein (1 µg/µL), respectively. These reactions were incubated under shaking conditions overnight (16 h) at 37 ± 2 °C and stored at −80 °C. Samples for Matrix Assisted Laser Desorption/Ionization Time-of-Flight (MALDI-TOF) MS analysis were prepared by desalting the reaction mixtures with C-18 Zip-Tip (Millipore, CA, USA). MALDI-TOF MS was performed at the Indian Institute of Science (IISc, Bangalore, India) Mass Spectrometry Facility, Department of Biological Sciences, Bangalore, using sinapic acid as the matrix.

#### 2.6.2. Raman Spectroscopy Analysis

A Raman alpha 300R (WITec, GmbH, Remscheid, Germany) confocal Raman microscope was used for acquiring spectra of purified protein sample. The equipment consisted of an excitation laser of 532 nm, 50X objective (Zeiss, NA = 0.8), 300 mm spectrograph equipped with 1200 gr/mm grating, and Thermo cooled charge-coupled detector (CCD). Raman-scattered photons were collected through a 50 µm fiber, and the spectra were obtained over the fingerprint region (600–1800 cm^−1^). The specimen was placed on a quartz window, and a total of 10 spectra were obtained at different locations. Each spectrum was acquired for 5 s and averaged over 10 accumulations. The highest peak normalization was performed using MATLAB (version R2021a). The mean spectrum was computed by averaging the intensities at the Y-axis and keeping the X-axis constant.

### 2.7. Sand-Packed Column Assay for Enhanced Oil Recovery

To evaluate the biosurfactant nature of recombinant iturin A, sand-packed column experiments were designed for improved oil recovery. Columns were made using 50 mL syringe tubes and were filled with 35 g of sand totally dried at 100 °C in a hot-air oven. Positive and negative controls were performed using SDS solution (1 mg/mL) and ultrapure water, respectively. Each column was flooded with 30 mL of 5% brine solution. The pore volume (PV) of the sand-packed column was calculated by determining the volume of the brine required to saturate the column. PV divided by the volume of the column indicates the porosity (%) of the sand-packed column. Crude oil was flooded into the sand-packed column till it replaced the whole volume of the brine. Hence, the oil in place (OOIP) indicates the volume of crude oil retained in the column [21].

Soi (%) = (OOIP/PV) × 100(1)

Swi (%) = ((PV − OOIP)/PV) × 100(2)

Residual oil saturation was obtained by flooding the brine into the column. Using the following equation, the oil recovered after flooding with the brine and the residual oil saturation was calculated.

Sor (%) = ((OOIP − Sorwf)/OOIP) × 100(3)

The remaining oil was flooded with recombinant iturin A and incubated at 50 ± 2 °C for 24 h. Recovered oil was used to identify the additional oil recovery.

AOR (%) = (Sorif/(OOIP − Sorwf)) × 100(4)

Where Sorwf in mL is residual oil saturation after water flooding, Sorif in mL is oil collected over residual oil saturation after iturin A flooding, Soi % is initial oil saturation, Swi in % is initial water saturation, Sor in % residual oil saturation and AOR in % represents additional oil recovery [22]. AOR % represents the enhanced oil recovery from the sand-packed column.

## 3. Results and Discussion

### 3.1. Genome Sequence Identification

The gene track of the *lpa-14* nucleotide sequence was identified and retrieved from NCBI and showed the product obtained from the gene (nucleotide accession no.: MN006821, protein ID: QHD44466.1), and the same was confirmed from the Norine database [23,24].

**Figure 1:**
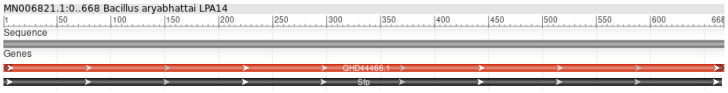
Graphical representation of the *lpa-14* (iturin synthesis gene) track with protein features and region features. Sfp-4′-phosphopantetheinyl transferase; QHD44466.1—protein ID (antimicrobial lipopeptide (iturin).

The same sequence was further used for the codon optimization, which was carried out using the Transeq tool. A codon-optimized sequence was further used for the cloning (Figure 1). The protein product translated from the nucleotide sequence was annotated as iturin A, which was considered to be a reference for the further process of cloning and expression through the vector.

### 3.2. Cloning into the pET-32A Vector

**Figure 2:**
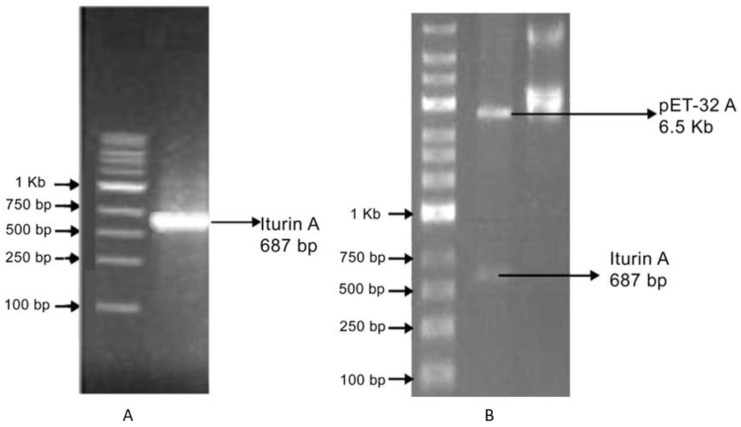
Cloning of the *lpa-14* gene from *Bacillus aryabhattai*: (**A**) amplification of the iturin A gene by PCR and (**B**) recombinant plasmid showing a band size of 687 bp (iturin A gene) and a band size of 6.5 Kb (pET-32A vector).

The coding region of the functional gene, iturin A, was amplified from the custom synthesized template DNA. The PCR product revealed a single band of 687 bp, as shown in Figure 2. PCR-amplified products of the gene were purified and cloned into the pET-32A vector and transformed. To confirm the presence of an insert, restriction digestion was performed with the help of BamHI and EcoRI. The transformed colonies were selected based on blue-white screening with colonies grown on ampicillin containing an agar plate. The coding sequences obtained from sequencing were then compared with the reference sequence in the GenBank with its orthologues for confirmation. Several factors, such as high production cost, downstream processing, and lower recovery rate of the biosurfactant, have triggered research on cloning biosurfactant genes to overcome these limitations. Cloning approaches can improvise the biosurfactant secretion capability of strains that can be efficiently utilized for oil recovery from reservoirs. Cloning aids to overcome the time-consuming biosurfactant screening procedures. Researchers have suggested the development and utilization of engineered strains for biosurfactant production with specific applications [25].

### 3.3. Expression and Purification of His–pET-32A-Iturin A Fusion Protein

**Figure 3:**
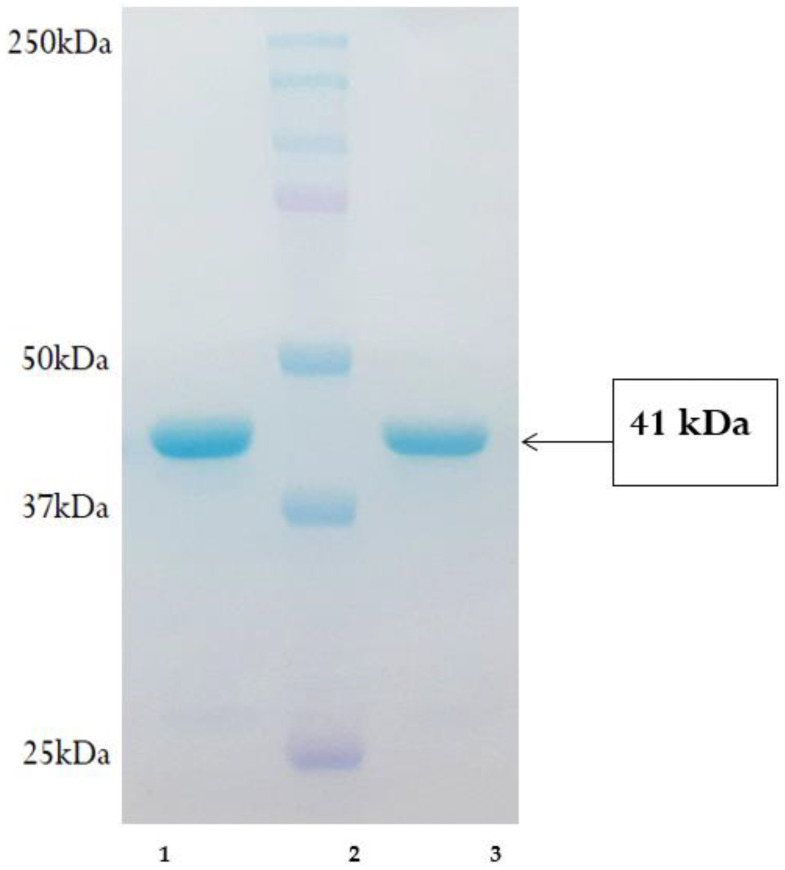
SDS-PAGE of recombinant iturin A. (**Lane 1**) Protein sample, (**Lane 2**) protein mol. wt marker (25 to 250 kDa), (**Lane 3**): protein sample.

The evolution of novel strains and the improvement of metabolites are both aided by genome shuffling. This method was utilized in an attempt to boost iturin A production by optimizing the codon. This approach aided in the high expression of recombinant iturin A, which possesses similar activity as a lipopeptide and surfactin. Hence, a highly expressed iturin A from *Bacillus aryabhattai* species was developed. The iturin A gene was composed of 687 bp with a conserved acyl transferase domain. The previous development of iturin A required cloning of a ~37 kbp gene cluster responsible for the biosynthesis of iturin A-like lipopeptides. Our attempt was to clone a functional fragment of partial iturin A, resulting in the increased production of the lipopeptide. This technique could be useful for creating microorganisms that operate as biological control agents. The expressed recombinant iturin A was purified on a nickel-NTA agarose column. Purified iturin A was further concentrated to 50 folds with the help of buffer exchange. SDS-PAGE (Figure S1) revealed the purity and molecular mass of iturin A with a fusion protein, which was observed to be 41 kDa (Figure 3). The yield of recombinant iturin A was found to be 60 g/L.

There was a 6.7-fold increase in production in comparison with wild iturin A, which yielded 8.86 g/L under optimized conditions (crude oil, 4.0%; yeast extract, 0.7%; NaNO_3_, 3.0%), as reported in our previous studies [23]. In a recent study [26], the recombinant strain HZ-PbacA showed high iturin A production owing to the replacement of the promoter PbacA in place of the promoter of the iturin A synthetase cluster. Additionally, an attempt was made to delete the regulator gene *abrB* to enhance the expression of iturin A synthetase by relieving the repression caused by AbrB on PbacA. As per previous studies, iturin A production from *B. subtilis* ZK0 and wild-type *B. subtilis* ZEB01 was found to be 0.005 g/L, which increased to 0.215 g/L by the simultaneous overexpression of the *comA* and *sigA* regulatory genes in *B. subtilis* ZK0 [27]. Dang et al. (2019) [28] reported 0.037 g/L of iturin A when a strong promoter was inserted into the upstream of itu operon for the transcription enhancement of iturin A genes. A further increase in yield (0.113 mg/L) was observed by the overexpression of the DegQ regulator. Several strategies for the overexpression of biosurfactant genes have been reviewed by Jimoh et al. (2021) [29] where the authors have reported the overexpression of signaling molecules that are involved in the regulation of biosynthetic clusters that trigger the production of a biosurfactant. For example, the overexpression of ComX and PhrC in *B. subtilis* revealed a 6.4-fold improvement in biosurfactant production. A high-level expression of molecules, such as biosurfactants and enzymes, is receiving importance as the functionality attributions of the molecules could be improved through this workflow [30].

### 3.4. Evaluation of Biosurfactant Activity

Recently, there has been great research interest in biosurfactants due to their exceptional properties, such as specificity, low toxicity, and eco-friendly characteristics. Biosurfactants have a wide range of applications in industries, such as food, medical, pharmaceutical, petrochemical, and agriculture. They have been demonstrated to have the ability to significantly decrease the surface tension and interfacial tension and form microemulsions [10].

**Figure 4:**
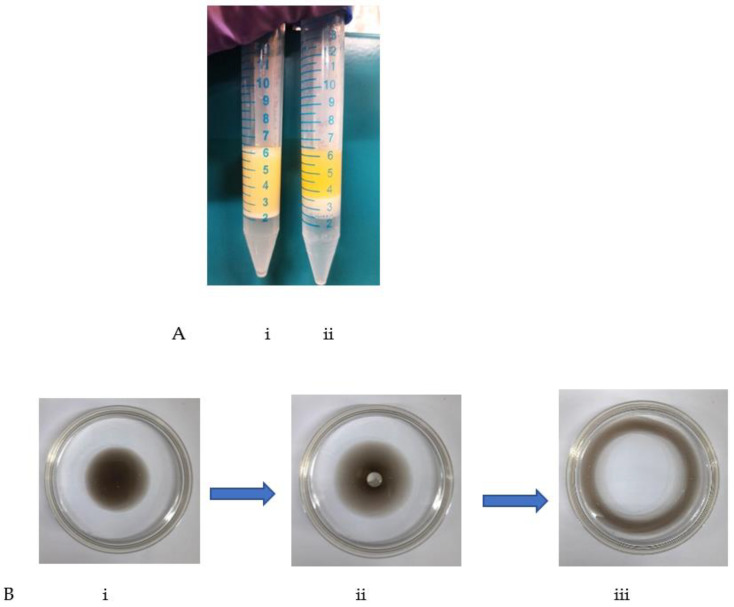
(**A**) Emulsification activity of recombinant iturin A: (i) test sample, (ii) negative control. (**B**) Oil spread assay: (i) motor oil before adding recombinant lipopeptide, (ii) recombinant lipopeptide added on oil surface, (iii) oil dispersion by recombinant lipopeptide.

The recombinant protein iturin A revealed biosurfactant activities based on a screening assay. The recombinant protein was evaluated for its emulsification index, which was found to be 74 ± 1.9% (Figure 4A). Additionally, the purified recombinant protein showed the dislocation of oil with a 2.2 cm diameter and an oil dispersion area of 29.88 ± 1.47 cm^2^ during the oil spread assay (Figure 4B). The oil dispersion and significant results of E24 prove the potential biosurfactant nature of recombinant iturin A. An E 24 higher than 30% is usually considered a significant emulsification activity.

In previous studies, a lipopeptide showed 68 ± 0.5% emulsification activity (E24) [7]. A cell-free broth of *B. velezensis* showed 56.2% E24 with engine oil [31]. A biosurfactant from mutant *A. niger* M2 showed 62.30% of E24 with 59.81 cm^2^ oil displacement area [32], and a 66.4 cm^2^ oil displacement area was shown by a biosurfactant from *R. arrhizus* [33]. In the present study, recombinant iturin A was revealed to possess potential biosurfactant activity and can be widely employed in oil recovery processes.

### 3.5. Analytical Characterization

#### 3.5.1. Mass Spectrometry Analysis

The digestion of protein was performed using the trypsin enzyme that cuts at the C-terminal of arginine (R) and lysine (K) [34]. The digestion was performed under two different conditions (protein: trypsin) of 50:1 and 25:1. The fragments were analyzed by MALDI-TOF MS. The MALDI-TOF spectra for both reactions showed the desired digested mass peaks for the protein. The predicted 16 mass fragments were computed with the iturin A primary protein sequence using the Expasy PeptideMass tool (https://web.expasy.org/peptide_mass, accessed on 15 October 2021). The mass spectra (Figure S2) showed 13 fragments for 50:1 reaction (Table 1), and [M + H]^+^ of iturin A at *m*/*z* 213.9814, *m*/*z* 627.2845, and *m*/*z* 594.3034 was not observed when compared with the predicted fragments, thus indicating 81.25% of protein sequence coverage. On the other hand, 14 fragments for 25:1 reaction were shown (Table 1), indicating 87.5% of the protein sequence coverage (a few lower molecular weight fragments were not observed). Following lower molecular weight fragments, [M + H]^+^ of iturin A at *m*/*z* 627.2845 and *m*/*z* 594.3034 was not observed; thus, the MALDI-TOF MS information for iturin A trypsin digestion confirms the identity of the iturin A protein. Previously, several workers reported the molecular masses and varied peaks for different class of peptides based on MALDI-TOF MS spectra. The potential iturin A class comprises different isomers ranging from iturin A_2_ to A_8_.

Ye et al. (2012) [35] reported the mass of purified iturin A_2_ to be 1042. The MALDI-TOF MS analysis of lipopeptides by Labiadh et al. (2021) [36] revealed a varied diversity of molecules, such as bacillomycin D, iturin C, fengycins, and kurstakin from *B. subtilis* strains. Secretion of lipopeptide isomers, such as iturin (majority), bacillomycin L, and fengycin, was confirmed by Stincone et al. (2020) [37] based on representative peaks (*m*/*z*) by mass spectrometry analyses. MALDI-TOF revealed the presence of majority of iturin molecules with fatty acid chain length of C_14_–C_17_ and a minor portion of fengycin A and B with a varying fatty acid chain length in the study conducted by Rodríguez-Chávez et al. (2019) [38].

#### 3.5.2. Raman Spectroscopy Analysis

**Figure 5:**
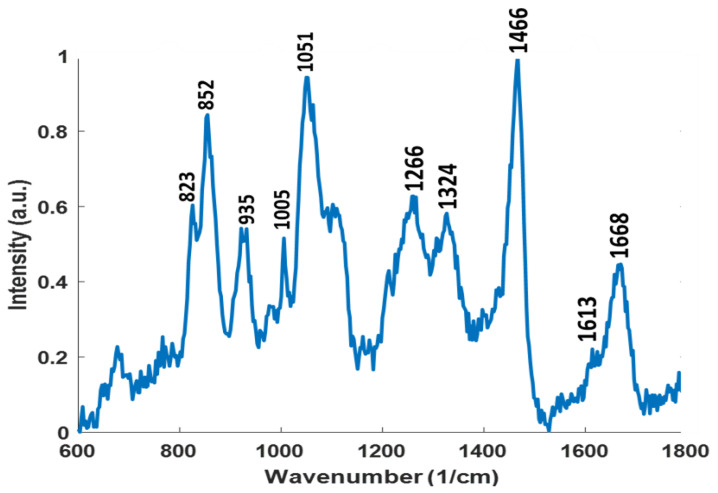
Mean Raman spectrum of purified recombinant iturin A.

Raman spectroscopic approaches have emerged as useful tools to analyze the composition and secondary structure of pure proteins. Spectral bands in a Raman spectrum are indicative of the presence of specific amino acids, protein structure, side-chain orientations, and local environments. As this method is not sensitive to water, it can be used to characterize materials in solution, preserving their biological activity. The average Raman spectrum shown in Figure 5 has discrete bands representing vibrational modes of the peptide backbone and its side chains. In addition to amide I (1668 cm^−1^) and III (1266 and 1324 cm^−1^) bands, amino-acid-specific Raman bands, such as 823 cm^−1^, 1613 cm^−1^ (ring breathing modes of tyrosine), 852 cm^−1^ (proline, hydroxyproline), 935 cm^−1^ (C–C stretching mode of proline and valine), 1005 cm^−1^ (phenylalanine), and 1051 cm^−1^ (C–N stretching), can be clearly seen. Spectral features in the 1050–1060 cm^−1^ area are suggestive of lipid presence [39] as these bands have been assigned to C–C stretching vibrations. This is further supported by the presence of a strong spectral band at 1466 cm^−1^ assigned to alkyl CH_2_ bends present in lipids. Raman spectroscopy has been employed by other researchers for several studies. Shi et al. (2021) [40] studied that a biosurfactant improved the CH_4_ hydrate structural stability by employing Raman spectra.

From the MALDI MS, the exact masses of the peptide fragments were obtained after trypsin digestion of iturin A. These masses match the predicted tryptic fragments obtained from the Expasy peptide mass bioinformatics tool using the primary sequence of iturin A (https://web.expasy.org/peptide_mass/, accessed on 15 October 2021). The observed masses (from two different tryptic digestion conditions) are within the accepted error range, which confirms the sequence/structure of iturin. On the other hand, from the Raman spectroscopic analysis, characteristic vibration/stretching bands were obtained, which are indicative of specific functional groups, such as amide bonds, certain amino acids (such as Tyr, Pro, and Val), and alkyl/lipid regions that provide preliminary information about the presence of a lipopeptide structure. Although, from the Raman analysis, the exact sequence of the protein cannot be obtained, the functional group identification from Raman data supports the MALDI MS analysis for deducing the structure of the protein based on its primary sequence (obtained from NCBI; https://www.ncbi.nlm.nih.gov/, accessed on 15 October 2021). Overall, the techniques are complementary and informative for understanding the structure of a protein [41,42].

**Figure 6:**
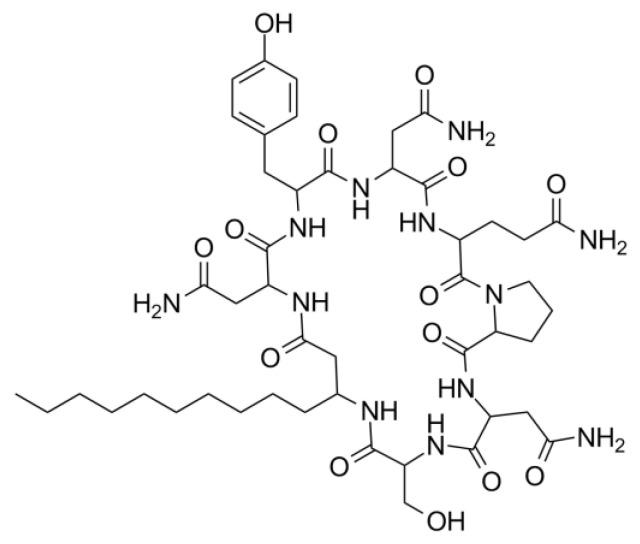
Two-dimensional structural representation of a biosurfactant (iturin A).

Iturin A is a lipopeptide (biosurfactant) that is a cyclic heptapeptide in nature [43] (Figure 6). The amino acid configuration is mainly Asx-Tyr-Asx-(Ser, Glx or Pro)-(Pro or Glu)-(Ser or Asn)-(Thr or Asn) in an LDDLLDL configuration sequence, which is closed by a beta-amino acid linking [44].

### 3.6. Application of a Biosurfactant for Enhanced Oil Recovery

The application of recombinant iturin A for oil recovery was evaluated a through-sand packed column (Figure S3). The column differs in structure and permeability due to its variation in PV and other parameters. The values of PV, OOIP, and Sorwf ranged from 9.88 to 10.08 mL, 6.92 to 7.18 mL, and 4.49 to 4.59 mL respectively. The residual oil was released by flooding recombinant iturin A into the sand-packed column. The quantity of oil recovered using recombinant iturin A after flooding with brine was 1.55 mL and was used to calculate AOR. Flooding sand-packed columns with recombinant iturin A yielded 61.18 ± 0.85% additional oil, as shown in Table 2. Compared with the negative control, it yielded 29% more oil.

The oil that is trapped in the pores of the sand-packed column imitates the natural oil trapped in the rock pores. Highly viscous residual oil and interfacial tension interferes with the flow properties of oils in wells [45]. The recovery of oil encompasses primary, secondary, and tertiary phases. The natural pressure drive method is used during the primary phase. In the secondary phase, the water flooding process is employed, which leads to the swelling of clay particles and causes subsequent damage to reservoirs. To combat this, brine solution is used during flooding. The application of surfactants is commonly evidenced in the tertiary phase of oil recovery [22]. The addition of a surfactant or biosurfactants exhibits the reduction of interfacial tension among the water and oil layer forming emulsion. It also varies the wettability attributes of the pore surfaces of the reservoir rocks and enhances the mobility of oil [46].

In a similar study conducted by Sharma et al. (2022) [47], 63% recovery of residual oil was observed using a biosurfactant from *Franconibacter* sp. IITDAS19. The brine solution is employed for enhancing the interactions between the oil and the biosurfactant following the theory of Derjaguin–Landau–Verwey–Overbeek (DLVO). Previously reported studies show that a biosurfactant produced from *Bacillus* sp. and *Fusarium* sp. BS-8, when used for oil recovery in a sand-packed column, resulted in 24.5% additional oil recovery [48]. A biosurfactant from *Pseudoxanthomonas* sp. G3 yielded 20% more oil recovery in comparison with the control in a sand-packed column [21]. Previous studies on oil recovery under simulated conditions reveal the mobilization of 17.15% residual oil by a glycoprotein N-4 biosurfactant [49]. A biosurfactant from *Candida tropicalis* MTCC 230 has also been reported for enhanced oil recovery [20]. A lipopeptide XT-2 biosurfactant was reported to recover 11–13% of residual oil [50]. The variability in the oil recovery could be due to the different types and concentration of biosurfactants. Recombinant iturin A demonstrated the recovery of a significant portion of residual oil, indicating its potential application in the petroleum industry in the long term.

## 4. Conclusions

The yield of iturin A was improvised (60 g/L) through the development of a recombinant lipopeptide by cloning, expression, and purification. The molecular mass of purified iturin A was 41 KDa. The recombinant protein was found to be a promising biosurfactant based on the oil spread assay and emulsification index (E 24). The identity of iturin A was confirmed with MALDI-TOF MS, and the structural elucidation of iturin A was carried out by Raman spectroscopy. Recombinant iturin A demonstrated enhanced oil recovery with a sand-packed column. This present study revealed a 6.7-fold increase in recombinant iturin A compared with that of our previous study, and it suggests real-time applications of a recombinant biosurfactant in oil reservoirs and the petroleum industry.

## Figures and Tables

**Table 1:** The predicted mass fragments and the observed mass fragments for 50:1 and 25:1 recombinant iturin A trypsin digestion reaction.

Predicted Masses, [M + H]^+^	Observed Masses in 50:1 Trypsin Digestion, [M + H]^+^	Observed Masses in 25:1 Trypsin Digestion, [M + H]^+^
3904.9	3904.2	3904.9
2135.9	-	2135.9
2095.9	2096.0	2095.9
1970.9	1970.9	1970.9
1886.0	1886.0	1886.0
1819.8	1819.8	1819.8
1532.7	1532.7	1532.7
1290.7	1290.8	1290.7
1133.4	1133.6	1133.5
1073.6	1073.6	1073.5
1031.6	1031.6	1031.6
937.4	937.4	937.4
767.3	767.4	767.4
627.3	-	-
623.3	623.3	623.3
594.3	-	-

**Table 2:** Enhanced oil recovery from the sand-packed column using recombinant iturin A.

Parameters	Sample	Positive Control	Negative Control
Pore volume (mL)	10 ± 0.8	9.97 ± 0.09	10 ± 0.08
Porosity (%)	20 ± 0.16	19.93 ± 0.18	20 ± 00.16
OOIP (mL)	7.1± 0.08	7 ± 0.08	7.03 ± 0.08
Soi (%)	71 ± 0.73	70.23 ± 0.41	70.34 ± 1.45
Swi (%)	29 ± 0.73	29.76 ± 0.41	29.65 ± 0.46
Sorwf (mL)	4.53 ± 0.04	4.55 ± 0.04	4.52 ± 0.02
Sor (%)	35.85 ± 0.58	35 ± 0.69	35.77 ± 0.75
Sorif (mL)	1.51 ± 0.04	1.05 ± 0.05	0.80 ± 0.03
AOR (%)	61.18 ± 0.85	42.85 ± 0.84	32.06 ± 1.04

## Data Availability

Not applicable.

## Supplementary Materials

The following supporting information can be downloaded at: https://www.mdpi.com/article/10.3390/gels8070403/s1, Figure S1: SDS-PAGE of recombinant iturin A; Figure S2: Maldi MS Spectra for (A) 50:1 trypsin digestion of protein and (B) 25:1 trypsin digestion of recombinant iturin A; Figure S3: Setup for enhanced oil recovery using recombinant iturin A form the sand packed column.
