# Supplementary material for: Gene Expression and Characterization of Iturin A Lipopeptide Biosurfactant from Bacillus aryabhattai for Enhanced Oil Recovery
Source: Gels. 2022 Jun 25;8(7):403. doi: 10.3390/gels8070403 (PMC9319305; doi:10.3390/gels8070403)
Supplement: Supplementary file 1 [file gels-08-00403-s001.zip › gels-1765234-supplementary.pdf]

Supplementary Information

Gene Expression and Characterization of Iturin A Lipopeptide Biosurfactant from *Bacillus aryabhattai* for Enhanced Oil Recovery

Deepak A. Yaraguppi [1], Zabin K. Bagewadi [1,*], Nilkamal Mahanta [2,*], Surya P. Singh [3], T. M. Yunus Khan [4], Sanjay H. Deshpande [1], Chaitra Soratur [1], Simita Das [2] and Dimple Saikia [3]

1 Department of Biotechnology, KLE Technological University, Hubballi, Karnataka 580031, India; deepak.yaraguppi@gmail.com (D.A.Y.); sanjay.deshpande2389@gmail.com (S.H.D.); chaitrasoratur@gmail.com (C.S.)

2 Department of Chemistry, Indian Institute of Technology, Dharwad 580011, India; 203041001@iitdh.ac.in

3 Department of Biosciences and Bioengineering, Indian Institute of Technology Dharwad 580011, India; ssingh@iitdh.ac.in (S.P.S.); 202041002@iitdh.ac.in (D.S.)

4 Department of Mechanical Engineering, College of Engineering, King Khalid University, Abha 61421, Saudi Arabia; yunus.tatagar@gmail.com

* Correspondence: zabinb@gmail.com (Z.K.B.); neel@iitdh.ac.in (N.M.); Tel.: +91-0836-2378231 (Z.K.B.); Fax: +91-0836-2374985 (Z.K.B.)

Supporting Information: 3 Pages, 3 Figures

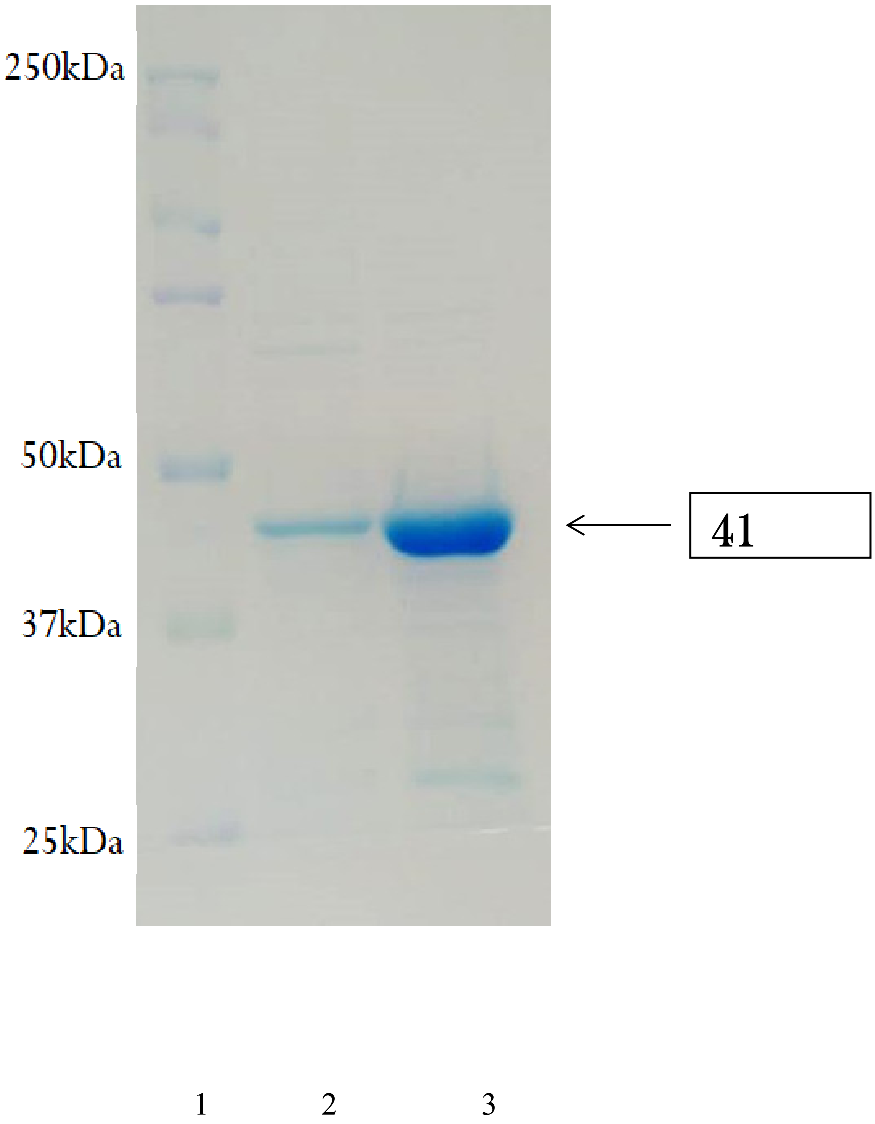

Figure S1. SDS-PAGE of recombinant iturin A. Lane 1= protein marker, Lane 2 = purified protein and Lane 3 = crude protein.

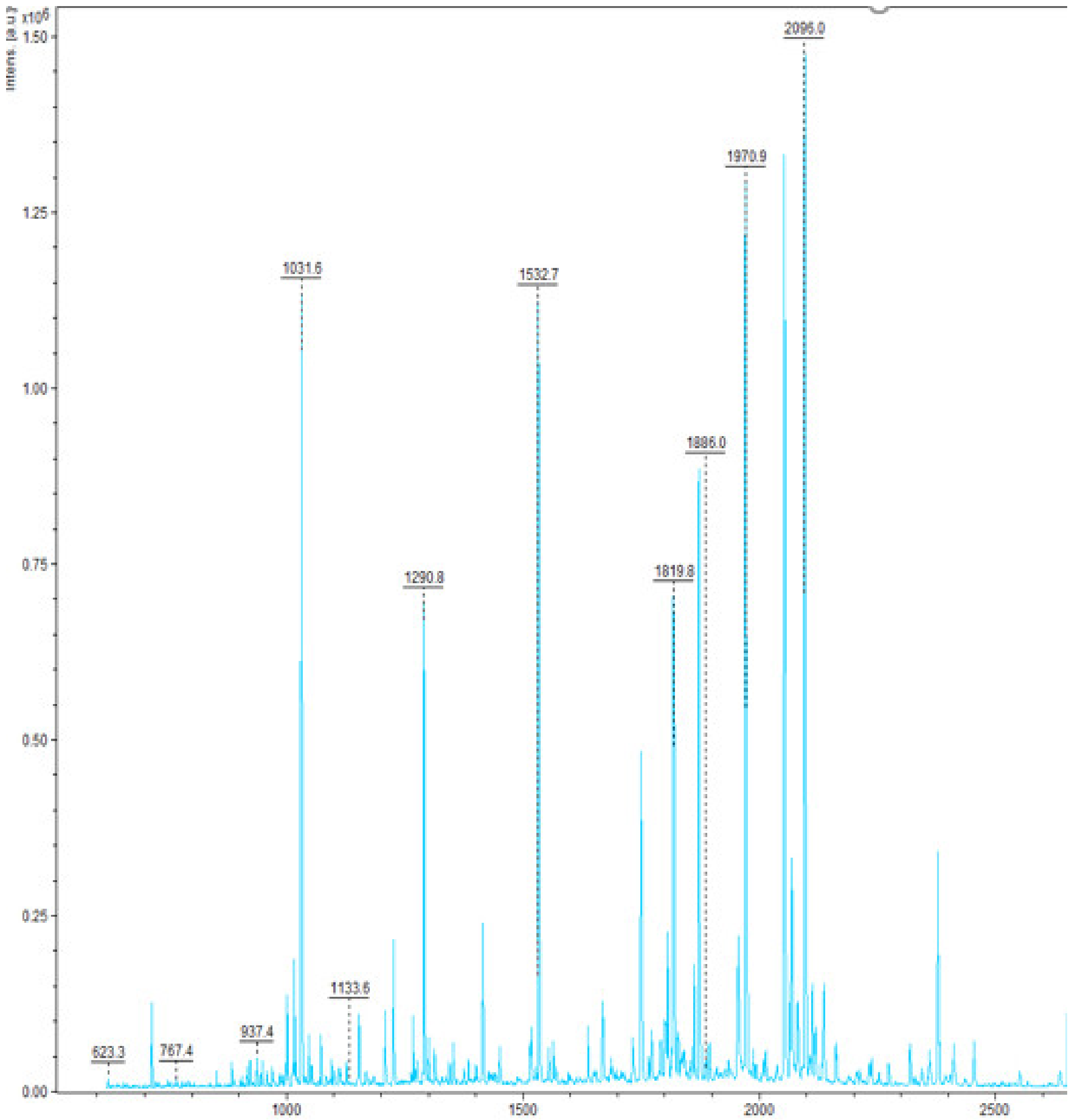

Intens. [a.u.]
x10^6
1.50
1.25
1.00
0.75
0.50
0.25
0.00
2096.0
1970.9
1031.6
1532.7
1886.0
1290.8
1819.8
1133.6
937.4
623.3
767.4
1000
1500
2000
2500

(A)

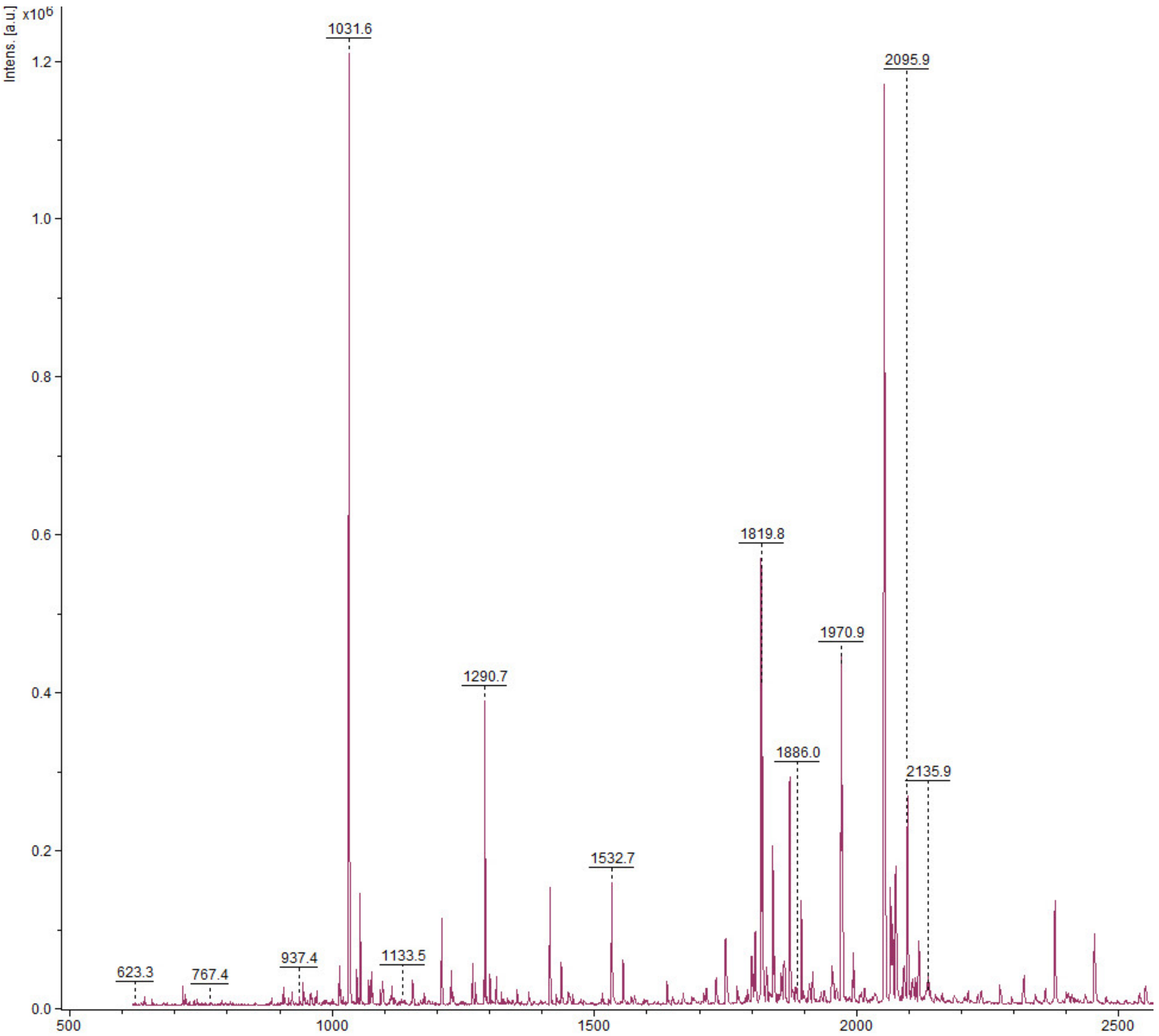

(B)

Figure S2. MALDI MS spectra for (A) 50:1 trypsin digestion of protein and (B) 25:1 trypsin digestion of recombinant iturin A.

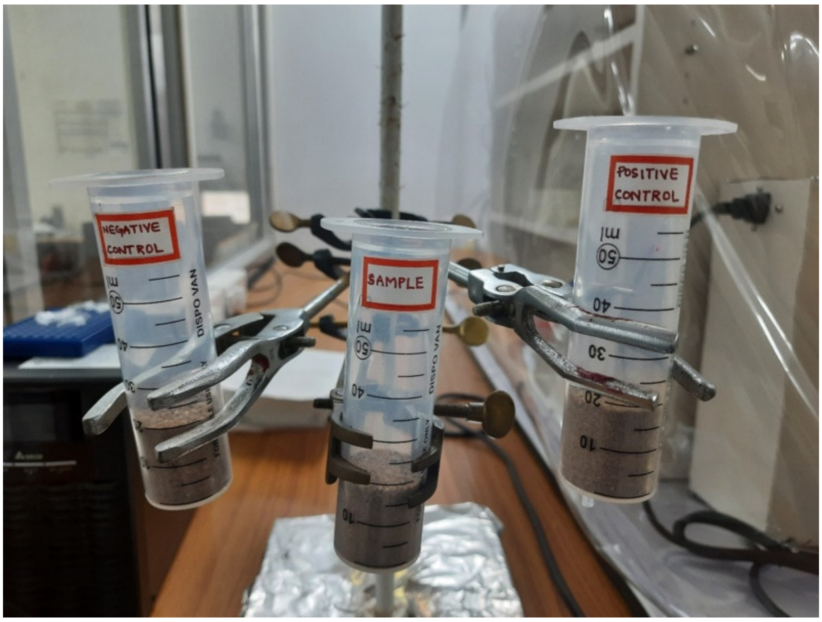

Figure S3. Setup for enhanced oil recovery using recombinant iturin A form the sand packed column
